# Design and Performance Evaluation of a Single-Phase Driven Ultrasonic Motor Using Bending-Bending Vibrations

**DOI:** 10.3390/mi12080853

**Published:** 2021-07-21

**Authors:** Dongmei Xu, Wenzhong Yang, Xuhui Zhang, Simiao Yu

**Affiliations:** 1 Shaanxi Key Laboratory of Mine Electromechanical Equipment Intelligent Monitoring, Xi’an University of Science and Technology, Xi’an 710054, China; ywz18503483941@163.com (W.Y.); zhangxh@xust.edu.cn (X.Z.); 2 School of Mechanical and Electrical Engineering, Xi’an University of Architecture and Technology, Xi’an 710055, China

**Keywords:** ultrasonic motor, single-phase driven, bending vibration

## Abstract

An ultrasonic motor as a kind of smart material drive actuator has potential in robots, aerocraft, medical operations, etc. The size of the ultrasonic motor and complex circuit limits the further application of ultrasonic motors. In this paper, a single-phase driven ultrasonic motor using Bending-Bending vibrations is proposed, which has advantages in structure miniaturization and circuit simplification. Hybrid bending vibration modes were used, which were excited by only single-phase voltage. The working principle based on an oblique line trajectory is illustrated. The working bending vibration modes and resonance frequencies of the bending vibration modes were calculated by the finite element method to verify the feasibility of the proposed ultrasonic motor. Additionally, the output performance was evaluated by experiment. This paper provides a single-phase driven ultrasonic motor using Bending-Bending vibrations, which has advantages in structure miniaturization and circuit simplification.

## 1. Introduction

The ultrasonic motor is a kind of special motor based on the inverse piezoelectric effect, which has the merits of no electromagnetic interference, no need for lubrication, fast response, and high positioning accuracy [1,2,3]. Thus, the ultrasonic motor has been used in fields such as camera lens drives, robots, optical fiber connections, biomedical engineering, etc. [4,5,6].

From the viewpoint of the phase number of excitation power supply, the ultrasonic motor can be divided into single-phase driven ones [7,8,9], two-phase driven ones [10,11], and multi-phase driven ones [12,13]. As the phase shift of each phase of excitation voltages should be adjustable, the power supplies of the two-phase-driven and multi-phase-driven ultrasonic motors are relatively complex and large. Tian et al. proposed a single-phase-driven piezoelectric actuator, which worked with an eight-shaped trajectory, and the piezo rings were clamped between the flange bolt and the horn [8]. A single-phase-driven piezoelectric actuator using the longitudinal bending coupling mode was proposed by Liu et al.; when one signal voltage with the frequency of the first longitudinal and third bending resonance frequency was applied to the motor and the boundary was unsymmetrical, oblique elliptical movement was generated to push the mover [14]. However, the consistency demand of the frequency of this longitudinal bending hybrid mode is relatively high. Flueckiger et al. proposed a single-phase ultrasonic motor, in which the longitudinal vibration mode was converted to the particular deformation of the resonator [15]. However, to obtain the forward motion, a signal frequency of 84 kHz was utilized, and to achieve the backward motion, a signal frequency of 69 kHz was used; thus, the output mechanical characteristics of the bi-directional motions are not consistent.

Based on the structure of the metal base and piezoelectric element, ultrasonic motors can be divided into two types: bonded type [16,17,18] and sandwich type [19,20,21]. The sandwich type ultrasonic motor has the advantages of large output force and high velocity. The sandwich type Langevin transducer in the literature [19] had an output mechanical force of 92 N and a no-load velocity of 0.47 m/s. A frog-shaped sandwich type piezoelectric actuator in the literature [20] achieved a maximum speed and a thrust of 287 mm/s and 11.8 N, respectively. However, because of the existence of stud structure, the structure of the sandwich type ultrasonic motor is relatively large. Therefore, in some specific situations, the use of the sandwich type ultrasonic motor is restricted.

In view of the above situations, a novel single-phase-driven bonded type ultrasonic motor is proposed in this study, which is beneficial to the miniaturization of motor size and drive circuit. Bending-Bending vibrations are utilized to form the desired oblique line driving trajectory. Additionally, there is no need for frequency degeneracy of Bending-Bending vibrations in this study. Section 2 introduces the structure and working principle of the single-phase-driven ultrasonic motor. Finite element analysis of the single-phase-driven ultrasonic motor is illustrated in Section 3. Output performance of this single-phase-driven bonded type ultrasonic motor is evaluated in Section 4. Finally, the conclusion is provided.

## 2. Structure and Working Principle of the Single-Phase-Driven Ultrasonic Motor

**Figure 1:**
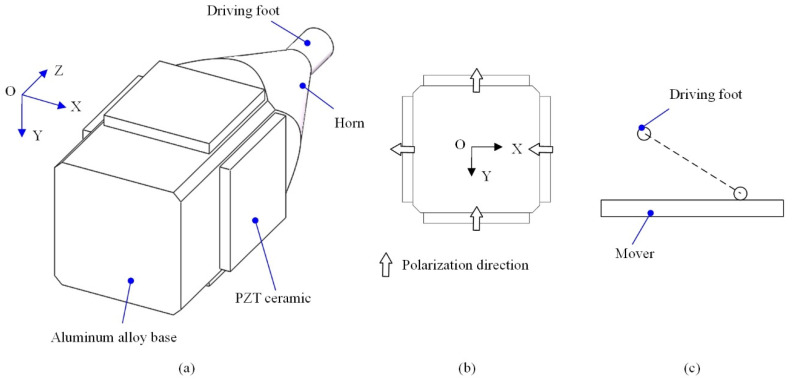
Structure and working principle of the proposed single-phase-driven ultrasonic motor: (**a**) structure, (**b**) polarization directions, (**c**) schematic diagram of driving trajectory.

The structure of the proposed single-phase-driven ultrasonic motor is shown in Figure 1a, which is composed of one aluminum alloy base and four pieces of PZT ceramic. The integrated base has three functioning parts, which are the base, horn, and driving foot. The horn is designed to magnify the vibration amplitude. In order to demonstrate the two orthogonal bending vibration modes of the ultrasonic motor, the polarization direction of four pieces of PZT ceramic is illustrated in Figure 1b. The bonded type of the proposed ultrasonic motor makes it suitable for miniaturization.

In addition, in a traditional case of a hybrid of two orthogonal bending vibration modes, two sinusoidal excitation voltages with a phase shift of 90 degrees are used to form an elliptical driving trajectory [22,23]. In this study, two orthogonal bending vibration modes with a 0-degree phase shift are utilized; thus, displacements in the OX and OY directions will be generated simultaneously; then the oblique line driving trajectory is formed, as shown in Figure 1c. Under the proposed principle, only single-phase excitation voltage is needed, which is beneficial to reduce the power cost, simplify the circuit, and miniaturize the whole ultrasonic motor.

## 3. Finite Element Analysis of the Single-Phase-Driven Ultrasonic Motor

**Figure 2:**
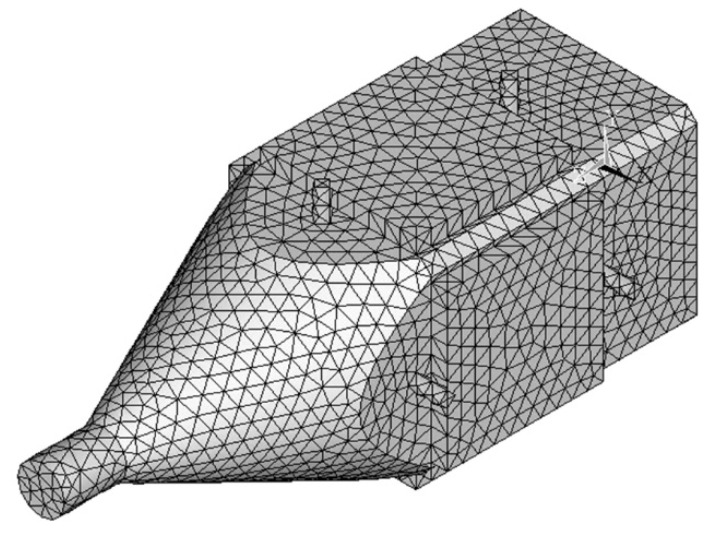
The FEM model of the proposed ultrasonic motor built in ANSYS.

The finite element method is used to calculate the vibration modes and to obtain the resonance frequencies of the Bending-Bending vibration modes. The finite element method (FEM) model of the proposed ultrasonic motor was built in ANSYS, as shown in Figure 2. The numbers of nodes and elements of the FEM model are 58657 and 40698, respectively. The element type of the FEM model is SOLID227. The properties of the aluminum alloy and the PZT ceramics are listed in Table 1.

**Figure 3:**
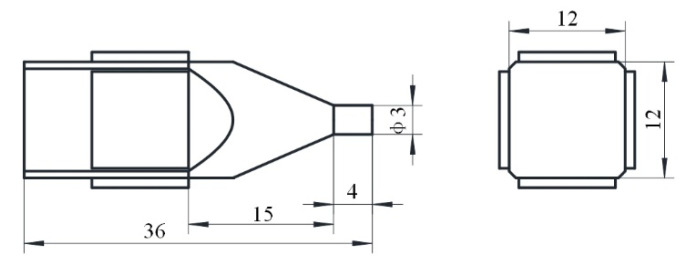
The dimensions of the proposed ultrasonic motor (unit: mm).

The optimized size of the proposed ultrasonic motor is achieved by parameter sensitivity analysis to ensure that the working frequency of the motor is greater than 20 kHz and the amplitude of bending vibration is greater than 1 μm. The total length of the aluminum alloy base is 36 mm, the height of the cross section of the base is 12 mm, the diameter of the driving foot is 3 mm, and the length of the horn is 15 mm. The size of the PZT ceramic is 10 × 10 × 1 mm^3^, and the position of the PZT ceramic is shown in Figure 3. The detailed dimensions of the single-phase-driven ultrasonic motor using Bending-Bending vibrations are shown in Figure 3.

**Figure 4:**
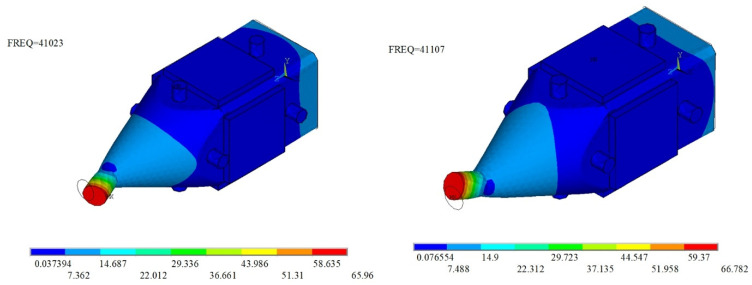
Bending vibration modes in OX and OY directions.

**Figure 5:**
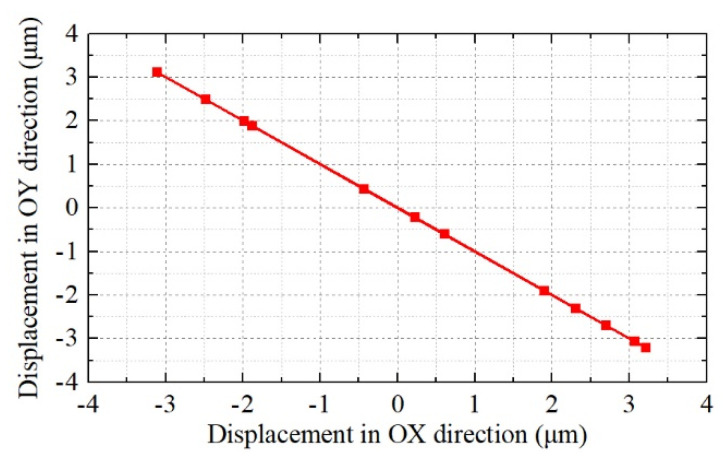
The calculated driving trajectory.

The calculated bending vibration modes in OX and OY directions are shown in Figure 4, the resonance frequencies are 41,023 Hz and 41,107 Hz, respectively, and the main reason for the frequency deviation is the unsymmetrical mesh of the model. The calculated driving trajectory is shown in Figure 5, which is an oblique line as proposed in Section 2. In addition, a clamping device was designed, which is shown in Section 4; in the FEM model, a displacement constraint was applied to the ultrasonic motor by cylinders to simulate the constraint of the clamping device.

## 4. Mechanical Characteristics of the Single-Phase-Driven Ultrasonic Motor

**Figure 6:**
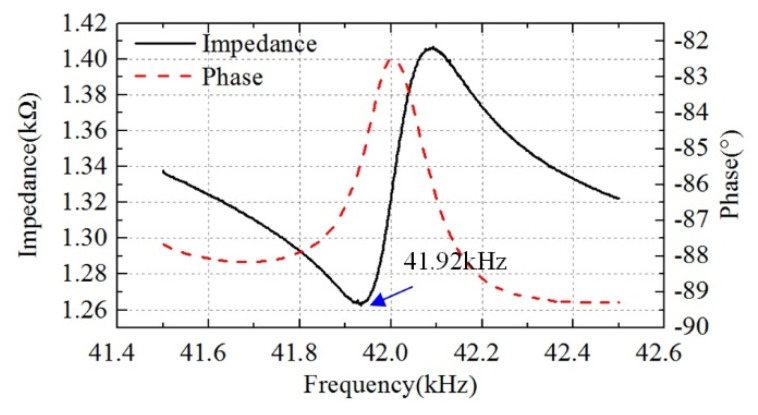
The impedance characteristics.

In order to evaluate the output performance, a prototype was manufactured. The stator of the ultrasonic motor was composed of one integrated aluminum alloy base and four pieces of PZT ceramic, the dimensions of which were the same as the optimized simulation results. Additionally, four PZT ceramics were pasted on the stator surface with resin glue at the positions shown in Figure 3, and the curing time was 24 h under the action of preload. The impedance characteristics were tested by an impedance analyzer (ZX80A, Zhixin Precision Electronics Co., Ltd., Changzhou, China), as shown in Figure 6. The tested resonance frequency was 41.92 kHz, and the deviation of the simulation resonance frequency and the test one was 855 Hz, which was approximately 2.1% of the simulation resonance frequency. The main reasons for the deviation are the parameters error of the aluminum alloy base and the PZT ceramics, the manufacturing error, and the error caused by the test condition of the impedance analyzer.

**Figure 7:**
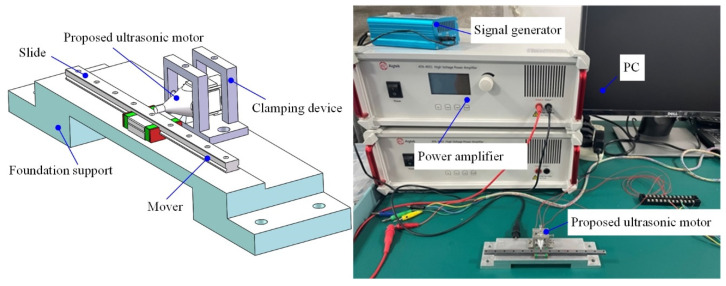
The clamping device and the experimental setup.

Then, the output performance was tested under the single-phase excitation voltage. The clamping device and the experimental setup are shown in Figure 7. The prototype was clamped and fixed on the foundation support; the driving foot was pressed on the linear guide rail. The excitation voltage was generated by the signal generator, then amplified by s power amplifier (ATA-4051, Agitek, China); the single-phase excitation voltage, sine signal, was applied to the PZT ceramics of the proposed ultrasonic motor.

**Figure 8:**
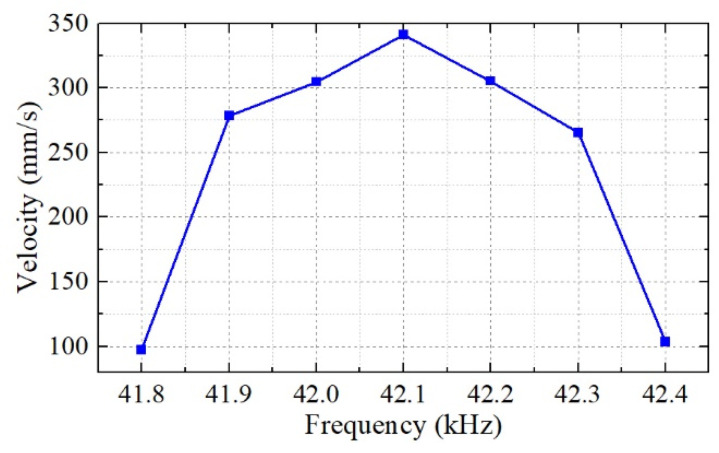
The output velocity versus the input excitation voltage frequency.

The output velocity versus the input excitation voltage frequency is shown in Figure 8. We can see that changing the frequency is another way to change the output velocity. The maximum output velocity of the mover was achieved at a frequency of 42.1 kHz. As the ultrasonic motor works in a resonance state, when the working frequency is far from the resonance frequency, the output velocity decreases rapidly.

**Figure 9:**
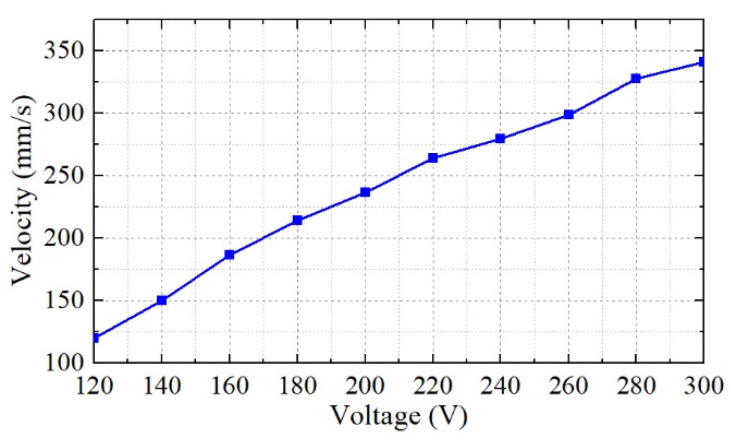
The output velocity versus the input excitation voltage amplitude.

The output velocity versus the input excitation voltage amplitude is shown in Figure 9, which indicates that we can change the voltage amplitude to increase the output velocity. With the excitation voltage no more than 120 V, the mover cannot be driven. The maximum velocity was approximately 340 mm/s under an excitation voltage of 300 V and 42.1 kHz.

In addition, the proposed single-phase ultrasonic motor using Bending-Bending vibration modes is feasible, which can also output rotary motion if the linear guide rail is replaced by a ring. The proposed bonded-type single-phase-driven ultrasonic motor not only has the merit of easy miniaturization, but also has a simple and easy miniaturization circuit. This single-phase-driven ultrasonic motor is indeed an impact motor, which has potential to be used in a high-accuracy platform.

## 5. Conclusions

A single-phase-driven ultrasonic motor using Bending-Bending vibrations was proposed in this paper. The structure of this ultrasonic motor was composed of a metal base and four pieces of PZT ceramic. Additionally, orthogonal bending vibration modes were excited simultaneously by only single-phase voltage, thus an oblique line driving trajectory was formed to drive the mover. The working principle was verified by the finite element method. Additionally, the impedance characteristics of the ultrasonic motor were tested. The output performance was evaluated by experiment. Additionally, the maximum output velocity under 300 V_p-p_ was 340 mm/s. The practicability of this proposed single-phase-driven ultrasonic motor was verified. This paper provides a single-phase-driven ultrasonic motor, which has merits in the miniaturization of structures and power circuits. In future work, we will focus on the verification of the linear trajectory and its application in a high-accuracy platform.

## Figures and Tables

**Table 1:** The properties of the aluminum alloy and the PZT ceramics.

PZT41	Aluminum Alloy
Piezoelectric matrix $d=\left[\begin{array}{cccccc} 0 & 0 & 0 & 0 & 5 & 0\\ 0 & 0 & 0 & 5 & 0 & 0\\ -1.6 & -1.6 & 3.3 & 0 & 0 & 0 \end{array}\right]\times 10^{-10}C/N$	Density *ρ* = 2810 kg/m^3^
Stiffness matrix $c^{E}=\left[\begin{array}{cccccc} 15 & 8.4 & 6.8 & 0 & 0 & 0\\ 8.4 & 15 & 6.8 & 0 & 0 & 0\\ 6.8 & 6.8 & 12.9 & 0 & 0 & 0\\ 0 & 0 & 0 & 3.3 & 0 & 0\\ 0 & 0 & 0 & 0 & 2.8 & 0\\ 0 & 0 & 0 & 0 & 0 & 2.8 \end{array}\right]\times 10^{10}{N/m}^{2}$	Poisson’s ratio *μ* = 0.33
Dielectric matrix $\varepsilon^{T}=\left[\begin{array}{ccc} 8.1 & 0 & 0\\ 0 & 8.1 & 0\\ 0 & 0 & 6.7 \end{array}0\right]\times 10^{-9}F/m$	Modulus of elasticity *E* = 4.72 GPa
